# Chronic Pulmonary Aspergillosis in Post Tuberculosis Patients in Indonesia and the Role of LDBio *Aspergillus* ICT as Part of the Diagnosis Scheme

**DOI:** 10.3390/jof6040318

**Published:** 2020-11-27

**Authors:** Anna Rozaliyani, Harmi Rosianawati, Diah Handayani, Heidy Agustin, Jamal Zaini, Ridhawati Syam, Robiatul Adawiyah, Mulyati Tugiran, Findra Setianingrum, Erlina Burhan, Chris Kosmidis, Retno Wahyuningsih

**Affiliations:** 1 Department of Parasitology, Faculty of Medicine, Universitas Indonesia, Jakarta 10430, Indonesia; ridhawatia@yahoo.com (R.S.); bundaadah@gmail.com (R.A.); dramulyati@yahoo.co.id (M.T.); findra.s88@gmail.com (F.S.); retnet2002@gmail.com (R.W.); 2 Pulmonary Mycosis Centre, Jakarta 10430, Indonesia; harmirosi520@gmail.com (H.R.); diahzulfitri@yahoo.com (D.H.); heidy_agst@yahoo.com (H.A.); jamalzaini@gmail.com (J.Z.); erlina_burhan@yahoo.com (E.B.); 3 Grha Permata Ibu Hospital, Depok 16425, Indonesia; 4 Department of Pulmonology and Respiratory Medicine, Faculty of Medicine, Universitas Indonesia, Persahabatan National Respiratory Referral Hospital, Jakarta 13230, Indonesia; 5 Manchester Academic Health Science Centre, Division of Infection, Immunity and Respiratory Medicine, School of Biological Sciences, Faculty of Biology, Medicine and Health, University of Manchester, Manchester M23 9LT, UK; chris.kosmidis@manchester.ac.uk; 6 Department of Parasitology, Faculty of Medicine, Universitas Kristen, Jakarta 13530, Indonesia

**Keywords:** chronic pulmonary aspergillosis, tuberculosis, Indonesia

## Abstract

Chronic pulmonary aspergillosis (CPA) is a common sequela of pulmonary tuberculosis (TB). The diagnosis of CPA is difficult and often misdiagnosed as smear-negative TB in endemic settings. *Aspergillus* IgG detection is the cornerstone of CPA diagnosis. There are a lack of studies on the prevalence of CPA in GeneXpert/smear-negative TB patients in Indonesia, despite a high number of TB cases. This study aims to determine the CPA rate in HIV-negative, GeneXpert-negative patients presenting with symptoms following completion of TB therapy and to evaluate the performance of LDBio *Aspergillus* immunochromatographic technology (ICT) lateral flow assay in the diagnosis of CPA. CPA was diagnosed on the basis of symptoms for ≥3 months, characteristic chest imaging and positive *Aspergillus* culture. Twenty (22%) out of 90 patients met the criteria for CPA. The LDBio test was positive in 16 (80%) CPA patients and in 21 (30%) non-CPA patients (*p* < 0.001) with 80% sensitivity and 70% specificity. Logistic regression revealed a positive LDBio *Aspergillus* ICT result, smoking history and diabetes to be important predictors of CPA diagnosis. Although CPA is an unrecognised disease in Indonesia, this study suggests that more than one in five GeneXpert negative patients with persistent symptoms following completion of TB therapy may have CPA.

## 1. Introduction

Aspergillosis can present as a chronic and progressive disease of the lung, called chronic pulmonary aspergillosis (CPA). This emerging disease affects more than 3 million people around the world, mainly survivors of pulmonary tuberculosis (TB) with estimates suggesting that more than 100,000 people develop CPA yearly as a consequence of TB [1,2]. The survival rate of CPA can be as low as 47% at 10 years depending on grade of lung damage and risk factors presence in patients [3]. The rate of CPA at the end of TB therapy in smear-negative, HIV-negative patients was 14.5% in a prior study in Nigeria [4].

Indonesia has one of the highest burdens of tuberculosis with estimated total TB incidence of 312 per 100,000 population in 2019 with high mortality rates (34 deaths per 100,000 HIV-negative TB population) [5,6]. Additionally, Indonesia also contributed to the global increase in newly diagnosed TB cases with a 69% increase from 2015 to 2019 [6]. Based on TB surveillance data of Indonesia, the total prevalence estimate of CPA in the country is ~83,000 patients with 17,561 new cases of CPA after pulmonary tuberculosis (PTB) every year [7]. A recent study showed 13% patients at the end of TB therapy in Indonesia developed CPA [8].

Detection of *Aspergillus* IgG in serum is the core of laboratory assessments for CPA [9,10,11]. Antibodies can be detected using various methods such as an enzyme-linked immunosorbent assay (ELISA), immunoprecipitation, complement fixation, haemagglutination, immunoblot and lateral flow assay [9,12]. Lateral flow assay (LFA) has been used in recent years to simplify the detection of *Aspergillus* IgG with fast results and minimal laboratory equipment [13,14]. LDBio *Aspergillus* immunochromatographic technology (ICT) is the only LFA available commercially for detection of *Aspergillus* IgG with excellent sensitivity (88.9–91.6%) and specificity (96.3–98%), but requires validation for the TB population [13,14]. Identification and treatment of CPA in low and middle-income countries is challenging mainly due to lack of awareness and diagnostics [15]. A point-of-care test would be a welcome development and would simplify diagnosis and management of CPA in Indonesia. Therefore, this study aimed to investigate the prevalence of CPA in GeneXpert-negative patients post TB in Indonesia and to validate the use of LDBio *Aspergillus* ICT for CPA diagnosis. 

## 2. Materials and Methods

Adults aged >18 years who presented to a respiratory clinic with symptoms after having completed TB therapy were recruited from Persahabatan National Respiratory Referral Hospital (Jakarta) and Grha Permata Ibu Hospital (Depok) from April 2019 to February 2020. Eligible patients had a negative GeneXpert and/or a negative acid-fast smear at the time of recruitment. The exclusion criteria were current or history of antifungal therapy in the previous month and HIV-positive status. LDBio *Aspergillus* immunochromatographic technology (ICT) lateral flow assay (LDBio Diagnostics, Lyon, France) was performed in all patients.

Ethical permissions were granted by The Ethics Committee of the Faculty of Medicine, Universitas Indonesia with reference number 25/UN2.F1/2019 and The Ethics Committee of Health Research of Persahabatan Hospital with reference number 11/KEPK-RSUPP/02/2019. Written informed consent was obtained from all the study participants. 

The main variables of this study were symptoms and their duration, serum LDBio *Aspergillus* ICT, fungal culture and full blood count. Clinical and chest imaging (chest X-ray and/or CT scan) information was collected concurrently with the sera. Chest imaging studies were evaluated by a radiologist blinded to clinical and serological findings.

CPA was diagnosed in patients with: (1) at least one of these symptoms including haemoptysis, cough, fatigue, chest pain and/or dyspnea >3 months, and (2) positive *Aspergillus* spp. culture from sputum, and (3) radiological appearances suggestive of CPA (at least one of cavitation and/or fungal ball confirmed by CT scan). The diagnostic criteria were modified from Denning et al. [16].

Data were reported using frequencies and percentages for categorical variables and mean with range for continuous variables. Fisher’s exact tests or chi-squared tests were applied for categorical variables. Student’s *t*-tests were used to detect differences between continuous variables. We selected variables with *p*-values of <0.2 and included these in the multivariate logistic regression model. Receiver operating curve (ROC) analyses were performed and area under curve (AUC) values, including 95% confidence intervals, were presented to assess the diagnostic ability of various combinations of risk factors of CPA and positive-LDBio *Aspergillus* ICT in relation to final CPA diagnosis using a combination of symptoms, radiology and culture as mentioned above. Sensitivity and specificity of LDBio *Aspergillus* ICT were also reported. SPSS version 25 (SPSS Inc, Chicago, IL, USA) was used throughout. 

## 3. Results

Of the 90 patients recruited for the study with a mean age of 51 years (range 18–80 years), 20 (22%) were classified as having CPA (Table 1). Median time from completion of TB therapy to recruitment was 7 (range: 1–360) months. The most common symptom was fatigue (48%, *n* = 43). The main radiological findings of the 90 patients were infiltrates (66%, *n* = 59), cavitation (61%, *n* = 55), bronchiectasis (29%, *n* = 26), and pleural thickening (28%, *n* = 25). Fourteen (16%) patients had hypertension as the most prevalent chronic disease.

**Figure 1:**
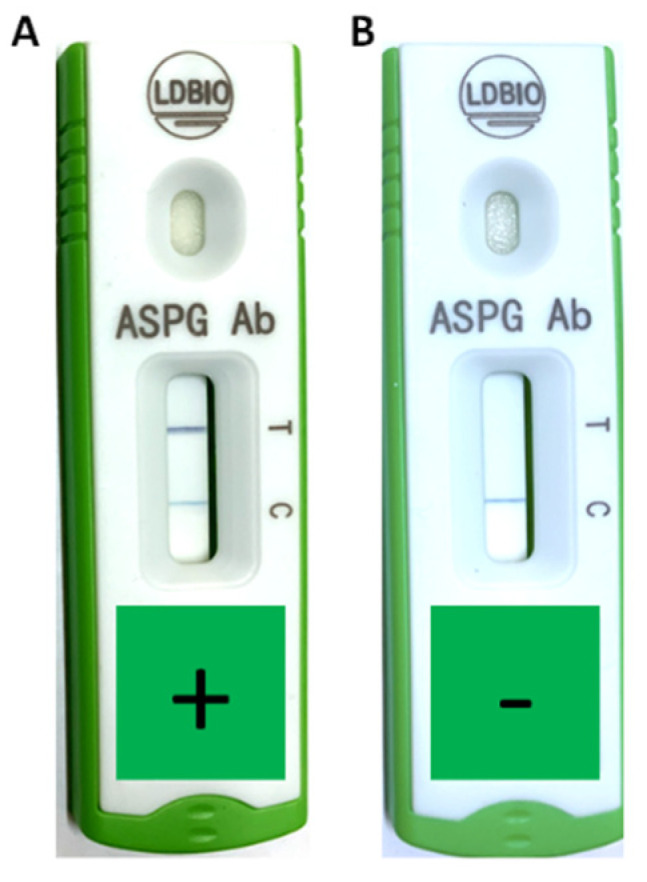
Positive (**A**) and negative (**B**) LDBio *Aspergillus* immunochromatographic technology in CPA patients from Indonesia. Positive result was indicated by gray/black line under “T” marker. The gray/black line under “C” marker was used for assured the validity of the test.

**Figure 2:**
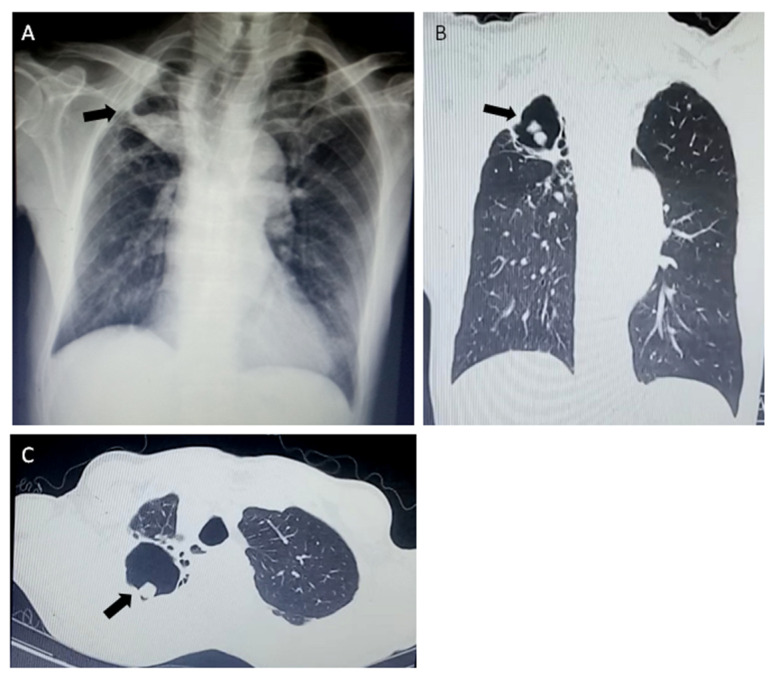
Radiological findings of chronic pulmonary aspergillosis (CPA) in a 51-year-old male patient two months after completion of TB treatment. A large cavity with aspergilloma (black arrows) of the left upper lobe in chest X-ray (**A**) and computed tomography scans (**B**,**C**).

LDBio *Aspergillus* ICT lateral flow assay was positive in 37 (41%) patients (Figure 1). The rate of positive *Aspergillus* culture from sputum was 47% (*n* = 42) (Table 2). The main symptoms in the CPA group were cough, haemoptysis and fatigue with 11 patients (55%) experiencing each of these symptoms. Cough (*p* = 0.020) and haemoptysis (*p* = 0.009) were significantly more common in the CPA group compared to the non-CPA group. CT of the thorax was performed in 55% (*n* = 11) of the CPA patients and chest X-ray was performed in all (*n* = 20) CPA patients. Thirty-three (47%) patients received a CT-scan and 66 (94%) patients had a chest X-ray from the non-CPA group. In particular, cavitation (*p* < 0.001), paracavitary fibrosis (*p* = 0.001), and aspergilloma (*p* = 0.021) were identified as more common in the CPA group compared to the non-CPA group. The radiological appearances of a patient with CPA are shown in Figure 2.

**Figure 3:**
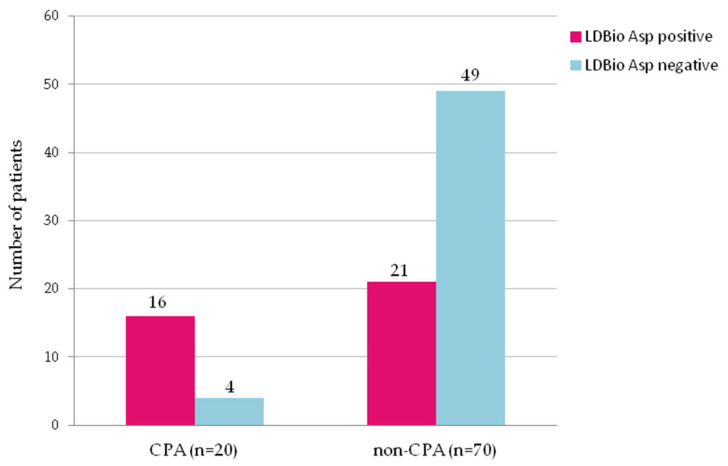
Rates of positivity for serum IgG-IgM *Aspergillus* antibody tested by LDBio *Aspergillus* immunochromatography in 20 patients with chronic pulmonary aspergillosis (CPA) and 70 non-CPA patients with history of pulmonary tuberculosis. The positivity rate in CPA group and in non-CPA group was 80% and 30%, respectively (*p* < 0.001).

The LDBio ICT was positive in 16 (80%) CPA patients and in 21 (30%) non-CPA patients (*p* < 0.001) (sensitivity: 80% (95%CI 62.4–97.5%); specificity: 70% (95%CI 59.3–80.7%)) (Figure 3). Three of four CPA patients with negative LDBio *Aspergillus* ICT had three months history of haemoptysis, while one patient experienced three months of coughing. All of these patients showed radiology appearances suggestive of CPA and positive *Aspergillus fumigatus* culture (two patients), *Aspergillus niger* (one patient), mixed colonies of A. fumigatus and *A. niger* (one patient) from sputum.

The distribution of the underlying chronic diseases differed between CPA and non-CPA in the proportion of diabetes mellitus cases. This disease was significantly (*p* = 0.022) more prevalent in the CPA group (30%) compared with the non-CPA group (9%). Fourteen (70%) patients from the CPA group had a past smoking history. This rate is significantly higher than the 32 (46%) patients with past smoking history in the non-CPA group. In contrast, the rates of other comorbidities, including hypertension, chronic obstructive pulmonary disease (COPD), asthma, pneumothorax, and lung malignancy were similar between the two groups. Furthermore, there were no significant differences in the body mass index (CPA: 18.7; non-CPA: 19.4; *p* = 0.465). The profile of blood count in the two groups also did not differ except for the hemoglobin level (CPA: 11.4 mg/L; non-CPA: 12.8 mg/L; *p* = 0.014). Seven (35%) CPA patients and eight (11%) non-CPA patients suffered from anemia (*p* = 0.036).

**Figure 4:**
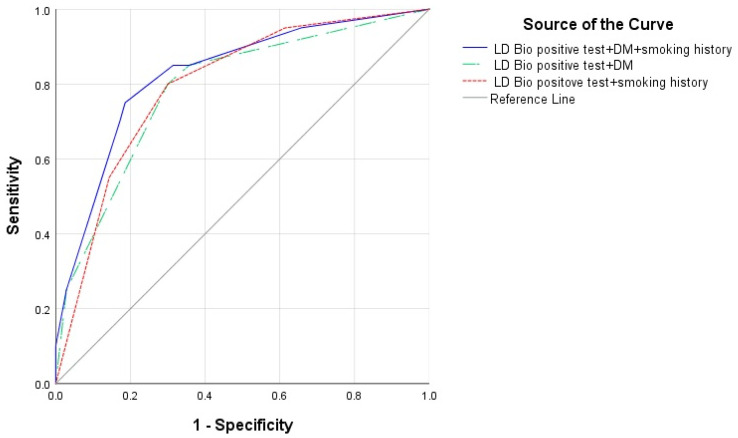
Receiver operating curve (ROC) of diagnosis prediction model of CPA using LDBio positive test + diabetes mellitus (DM) + smoking history (area under curve (AUC) 0.827, 95% CI 0.722–0.932), LD Bio positive test + DM (AUC 0.788, 95% CI 0.673–0.903) and LD Bio positive test + smoking history (AUC 0.796, 95% CI 0.690–0.903).

The following five variables were considered for selection in the logistic regression model for predicting diagnosis of CPA: LDBio positive test, diabetes mellitus, anemia, smoking history, and duration of past TB therapy. LDBio positive test (OR: 8.55, 95%CI 2.22–33.33, *p* = 0.002), diabetes mellitus (OR: 6.80; 95% CI 1.32–35.71, *p* = 0.022) and smoking history (OR: 34.48, 95% CI 1.18–21.74, *p* = 0.029) were revealed as risk factors for CPA. The ROC AUC for the prediction model using all three variables was 0.827 (95% CI 0.722–0.932). This ROC AUC score is the highest compared to the combination of the LDBio positive test and diabetes mellitus and the LDBio positive test and smoking history (Figure 4).

## 4. Discussion

In the present study, we show that more than one in five HIV-negative patients who present with ongoing symptoms and a negative GeneXpert test following completion of TB therapy may have CPA. This is in line with many published studies that indicate CPA as an important complication in post TB patients [2,4,17,18]. In a study from Uganda, 26% of patients with cavitation following TB treatment had CPA [2]. The percentage we observed is higher compared to that reported in patients just finishing TB therapy in Indonesia (13% in both positive and negative-smear TB), possibly indicating that CPA develops over time after completion of TB treatment [8]. The mean age in the current study was older than in a previous study [8] from Indonesia and might indicate the role of age as a risk factor for a poor outcome in TB [19]. Similarly, in Nigeria, 14.5% of patients who completed TB therapy had CPA [4]. The time of TB treatment until patients were recruited into the study was 7 months. The higher CPA rate in our study could also be attributed to the use of a CT-scan in half of the patients (49%, *n* = 44), which allowed high accuracy of cavity detection and monitoring the response of treatment compared to previous studies which mostly used a chest X-ray as their imaging modality [8,20]. Five patients (25%) would not have been identified as having CPA in this study if we had used only a chest X-ray for chest imaging screening because the X-ray failed to detect cavitation in these patients. This is the first study to reveal the prevalence of CPA in GeneXpert-negative post TB patients in Indonesia.

The diagnosis of CPA in Indonesia is hampered by lack of access to *Aspergillus* IgG detection, which plays a critical part as a diagnostic criterion for CPA [15,16]. The point-of-care test used in this study is suitable for resource-constrained settings such as Indonesia because of its ability to produce fast results with minimum laboratory equipment. To the best of our knowledge, this is the first evaluation of LDBio *Aspergillus* ICT performance in a TB endemic area and resource-constrained settings in Asia. A recent paper from Uganda reports successful routine use of the LDBio *Aspergillus* ICT [21]. The sensitivity and specificity of LDBio *Aspergillus* ICT is 88.9–91.6% and 96.3–98% for diagnosing CPA in a European population, a non-endemic TB area [13,14]. The test’s performance was comparable to the ImmunoCAP *Aspergillus* IgG test [13].

The LDBio *Aspergillus* ICT was used in our study to detect the *Aspergillus* IgG and IgM from sera of TB patients with 80% sensitivity and 70% specificity. An intrinsic difficulty in establishing the utility of a serological diagnostics in CPA is the insensitivity of culture [22]. Here, we used a positive culture as a diagnostic criterion, which yielded a sensitivity for LDBio *Aspergillus* ICT of 80%. There were 21 patients (23%) with presumed false positive results of LD Bio *Aspergillus* ICT, including nine patients with probable CPA (symptoms and radiology suggestive but culture negative). These nine patients might have false negative culture result due to insensitivity of the fungal culture. The quality and quantity of the sputum sample should be evaluated to optimise the fungal culture detection.

If all patients with 3 months of symptoms and radiology consistent with CPA only are used, the LDBio *Aspergillus* ICT sensitivity falls to 61%, and there are only 13% (*n* = 12) apparent false positives; the specificity improves slightly to 76%. The relatively low specificity of LDBio *Aspergillus* ICT could in part reflect early CPA disease, before it is radiologically apparent. Follow up of these cases is required to ascertain if they develop more overt features of CPA over the following weeks and months.

The difference in the performance of LDBio *Aspergillus* ICT may be explained by different characteristics of the population tested or a difference in species distribution of *Aspergillus*. *A. fumigatus* was the dominant *Aspergillus* species detected in CPA patients followed by *A. niger* and *A. flavus*. Previous studies from Africa and Asia demonstrated the same distribution of *Aspergillus* species among TB patients, with *A. fumigatus* most common but with significant presence *of A. niger* and *A. flavus* [4,23,24,25]. This is different from the culture result of CPA patients from the UK, which was dominated by *A. fumigatus* (91%) [13]. Two patients with false negative LDBio *Aspergillus* ICT in our study had positive culture of *A. niger* as a single colony and mixed colonies between *A. fumigatus* and *A. niger*. [13]. However, there was no significant difference in LDBio *Aspergillus* ICT performance between *A. fumigatus* and non-*A. fumigatus* CPA cases [13]. Further work is required to establish the cross-reactivity status between different species of *Aspergillus*, as well as the test’s performance in different patient populations. LDBio *Aspergillus* ICT detects both IgG and IgM. IgM is predicted to be present in 50% of CPA patients caused by *A. fumigatus*, which may not apply to other *Aspergillus* species [9,26].

Smoking history was an important risk factor for development of CPA in this study. This result reflects previous reports which also found that smoking was linked with pulmonary mycosis [25]. Smoking may accelerate the lung damage caused by TB, leading to a high risk of fungal infection [25,27,28]. Undiagnosed COPD, another well-described risk factor for CPA, may have been present in our patients. In addition, the current study revealed diabetes as a predictor for CPA diagnosis. Diabetes was the second most common non-pulmonary disease—after hypertension—found in CPA patients in Jakarta [8]. Diabetes was found to be the most commonly associated condition in CPA patients in Pakistan [29]. This is consistent with the literature that indicates increased rates of cavitation in the lung and death during TB therapy associated with diabetes [30,31]. Poorly controlled diabetes is also associated with subacute invasive aspergillosis, an aggressive form of CPA [32]. Diabetes may be linked to *A. niger* CPA as well, based on a study from Brazil [33].

The strength of this study is the exclusion of diagnosis of relapse of TB by a negative GeneXpert-TB in all patients [16]. GeneXpert-TB demonstrated high sensitivity (86.8%) and specificity (93.1%) for the TB detection test [34]. The limitation of our study is the small number of CPA cases. Additionally, comparison between LDBio *Aspergillus* ICT and other methods of *Aspergillus*-IgG detection test was not performed because of lack of availability of these tests in Indonesia. Finally, we do not provide information of long-term outcomes of these patients or on antifungal treatment; subsequent studies should focus on longitudinal follow up of CPA patients in Indonesia following antifungal therapy.

In conclusion, this study has identified that CPA is a common sequela in patients with prior TB presenting with symptoms and a negative GeneXpert-TB. LDBio *Aspergillus* ICT showed a good diagnostic performance and could be used in Indonesia due to the fast and simple procedures of the test. In patients with persistent symptoms following TB therapy and an abnormal chest X-ray, the combination of a positive LDBio *Aspergillus* ICT, smoking history and diabetes can be used as a useful predictor of CPA diagnosis while waiting for additional radiology and the fungal culture result. A combination of these clinical risk factors may assist the early screening of CPA and prioritise serological testing for this population. Future research should explore the diagnosis of CPA in a larger study comparing various modalities of serological diagnosis.

## Figures and Tables

**Table 1:** Patient characteristics.

Variables	ALL (*n* = 90)	CPA (*n* = 20)	Non CPA (*n* = 70)	*p*-Value
Gender				
Male	61 (68%)	13 (65%)	48 (69%)	
Female	29 (32%)	7 (35%)	22 (31%)	0.763
Age, mean (range)	51 (18–80)	50.7 (29–66)	51.1 (18–80)	0.898
Symptoms (≥3 months)				
Cough	30 (33%)	11 (55%)	19 (27%)	0.020
Haemoptysis	28 (31%)	11 (55%)	17 (24%)	0.009
Fatigue	43 (48%)	11 (55%)	32 (46%)	0.463
Dyspnoea	30 (33%)	5 (25%)	25 (36%)	0.370
Chest pain	17 (19%)	6 (30%)	11 (16%)	0.195
Radiology				
Infiltrates	59 (66%)	10 (50%)	49 (70%)	0.097
Cavitation	55 (61%)	20 (100%)	35 (50%)	<0.001
Air fluid level in cavities	3 (3%)	2 (10%)	1 (1%)	0.123
Paracavitary fibrosis	18 (20%)	10 (50%)	8 (11%)	0.001
Pleural thickening	25 (28%)	9 (45%)	16 (23%)	0.051
Nodules	16 (18%)	6 (30%)	10 (14%)	0.180
Bronchiectasis	26 (29%)	9 (45%)	17 (24%)	0.071
Aspergilloma	6 (7%)	4 (20%)	2 (3%)	0.021
Pleural effusion	23 (26%)	7 (35%)	16 (23%)	0.272
Chronic diseases				
Diabetes mellitus	12 (13%)	6 (30%)	6 (9%)	0.022
Hypertension	14 (16%)	3 (15%)	11 (16%)	1
Asthma	6 (7%)	1 (5%)	5 (7%)	1
Chronic pulmonary obstructive disease	10 (11%)	3 (15%)	7 (10%)	0.686
Pneumothorax	4 (4%)	1 (5%)	3 (4%)	1
Body mass index, mean (range)	19.3 (10.4–31.2)	18.7 (13.7–26.5)	19.4 (10.4–31.2)	0.465
Duration of TB treatment, mean (range), months	9 (6–26)	12.6 (6–26)	8 (6–20)	<0.001
TB treatment > 6 months	33 (37%)	11 (55%)	22 (31%)	0.054
Smoking history	46 (51%)	14 (70%)	32(46%)	0.055

**Table 2:** Laboratory results.

Variables	ALL (*n* = 90)	CPA (*n* = 20)	Non CPA (*n* = 70)	*p*-Value
LDBio *Aspergillus* positive	37 (41%)	16 (80%)	21 (30%)	<0.001
Culture positive *Aspergillus*	42 (47%)	20 (100%)	22 (31%)	<0.001
Only *Aspergillus*	13 (14%)	5 (25%)	8 (11%)	0.153
*Aspergillus* & *Penicillium*	4 (4%)	1 (5%)	3 (4%)	1
*Aspergillus* & *Candida*	20 (22%)	10 (50%)	10 (14%)	0.002
*Aspergillus*, *Penicillium* & *Candida*	5 (6%)	4 (20%)	1 (1%)	0.008
*Aspergillus* species distribution				
*Aspergillus fumigatus*	33 (37%)	15 (75%)	18 (26%)	<0.001
*Aspergillus niger*	20 (22%)	9 (45%)	11 (16%)	0.012
*Aspergillus flavus*	3 (3%)	1 (5%)	2 (3%)	0.534
Blood test				
Hemoglobin (g/dL)	12.5 (5–17.7)	11.4 (5–16.6)	12.8 (8.7–17.7)	0.014
Leukocyte (10^3^/µL)	11.4 (3.6–40)	12 (5.8–21.6)	11.5 (3.6–40)	0.668
Basophil (%)	0.4 (0–1.1)	0.5 (0.1–1.1)	0.3 (0–1)	0.133
Eosinophil (%)	1.8 (0–14.2)	2.3 (0–14.2)	1.7 (0–11.7)	0.364
Neutrophil (%)	77.6 (52.7–93.4)	75 (57.2–90.4)	78.2 (52.7–93.4)	0.245
Lymphocyte (%)	13.7 (2.8–36.9)	15.3 (3.9–31.9)	13.3 (2.8–36.9)	0.362
Monocyte (%)	6.4 (0.9–16.6)	6.9 (2.8–13.7)	6.3 (0.9–16.6)	0.399
Anemia (<11 g/dL)	15 (17%)	7 (35%)	8 (11%)	0.036

## References

[1] Brown G.D., Denning D.W., Gow N.A., Levitz S.M., Netea M.G., White T.C. (2012). Hidden Killers: Human Fungal Infections. Sci. Transl. Med..

[2] Page I.D., Byanyima R., Hosmane S., Onyachi N., Opira C., Richardson M., Sawyer R., Sharman A., Denning D.W. (2019). Chronic Pulmonary Aspergillosis Commonly Complicates Treated Pulmonary Tuberculosis with Residual Cavitation. Eur. Respir. J..

[3] Lowes D., Al-Shair K., Newton P.J., Morris J., Harris C., Rautemaa-Richardson R., Denning D.W. (2017). Predictors of Mortality in Chronic Pulmonary Aspergillosis. Eur. Respir. J..

[4] Oladele R.O., Irurhe N.K., Foden P., Akanmu A.S., Gbaja-Biamila T., Nwosu A., Ekundayo H.A., Ogunsola F.T., Richardson M.D., Denning D.W. (2017). Chronic Pulmonary Aspergillosis as a Cause of Smear-Negative TB and/or TB Treatment Failure in Nigerians. Int. J. Tuberc. Lung Dis..

[5] WHO (2020). Tuberculosis Profile: Indonesia. https://worldhealthorg.shinyapps.io/tb_profiles/?_inputs_&lan=%22EN%22&iso2=%22ID%22.

[6] WHO (2020). Global Tuberculosis Report: Executive Summary. https://www.who.int/docs/default-source/documents/tuberculosis/execsumm-11nov2020.pdf?sfvrsn=e1d925f_4.

[7] Wahyuningsih R., Adawiyah R., Rozaliyani A., Denning D.W., Prihartono J., Syam R., Wulandari E.A., Imran D., Tugiran M., Forman E. Estimation of the Serious Mycoses Burden in Indonesia. Proceedings of the 27th European Congress of Clinical Microbiology and Infectious Diseases 2017.

[8] Setianingrum F., Rozaliyani A., Syam R., Adawiyah R., Tugiran M., Sari C.Y.I., Burhan E., Wahyuningsih R., Rauteema-Richradson R., Denning D.W. (2020). Evaluation and Comparison of Automated and Manual ELISA for Diagnosis of Chronic Pulmonary Aspergillosis (CPA) in Indonesia. Diagn. Microbiol. Infect. Dis..

[9] Page I.D., Richardson M., Denning D.W. (2015). Antibody Testing in Aspergillosis--Quo Vadis?. Med. Mycol..

[10] Denning D.W., Cadranel J., Beigelman-Aubry C., Ader F., Chakrabarti A., Blot S., Ullmann A.J., Dimopoulos G., Lange C. (2016). Chronic Pulmonary Aspergillosis: Rationale and Clinical Guidelines for Diagnosis and Management. Eur. Respir. J..

[11] Richardson M., Page I. (2018). Role of Serological Tests in the Diagnosis of Mold Infections. Curr. Fungal Infect. Rep..

[12] Bongomin F., Asio L.G., Baluku J.B., Kwizera R., Denning D.W. (2020). Chronic Pulmonary Aspergillosis: Notes for a Clinician in a Resource-Limited Setting Where There Is No Mycologist. J. Fungi.

[13] Stucky Hunter E., Richardson M.D., Denning D.W. (2019). Evaluation of LDBio Aspergillus ICT Lateral Flow Assay for IgG and IgM Antibody Detection in Chronic Pulmonary Aspergillosis. J. Clin. Microbiol..

[14] Piarroux R.P., Romain T., Martin A., Vainqueur D., Vitte J., Lachaud L., Gangneux J.P., Gabriel F., Fillaux J., Ranque S. (2019). Multicenter Evaluation of a Novel Immunochromatographic Test for Anti-Aspergillus IgG Detection. Front. Cell. Infect. Microbiol..

[15] Rozaliyani A., Jusuf A., ZS P., Burhan E., Handayani D., Widowati H., Pratama S., Setianingrum F. (2019). Pulmonary Mycoses in Indonesia: Current Situations and Future Challenges. J. Respirologi Indones..

[16] Denning D.W., Page I., Chakaya J., Jabeen K., Jude C.M., Cornet M., Alastruey-Izquierdo A., Bongomin F., Bowyer P., Chakrabarti A. (2018). Case Definition of Chronic Pulmonary Aspergillosis in Resource-Limited Settings: Catalysing Research and Clinical Care. Emerg. Infect. Dis..

[17] Denning D.W., Pleuvry A., Cole D.C. (2011). Global Burden of Chronic Pulmonary Aspergillosis as a Sequel to Pulmonary Tuberculosis. Bull. World Health Organ..

[18] Kampen S.C., Van Wanner A., Edwards M., Harries A.D., Kirenga B.J., Chakaya J., Jones R. (2018). International Research and Guidelines on Post-Tuberculosis Chronic Lung Disorders: A Systematic Scoping Review. BMJ Glob. Health.

[19] Gennaro F., Di Vittozzi P., Gualano G., Musso M., Mosti S., Mencarini P., Pareo C., Caro A., Di Schinin V., Girardi E. (2019). Active Pulmonary Tuberculosis in Elderly Patients: A 2016–2019 Retrospective Analysis from an Italian Referral Hospital. Antibiotics.

[20] Godet C., Laurent F., Bergeron A., Ingrand P., Beigelman-Aubry C., Camara B., Cottin V., Germaud P., Philippe B., Pison C. (2016). CT Imaging Assessment of Response to Treatment in Chronic Pulmonary Aspergillosis. Chest.

[21] Kwizera R., Katende A., Teu A., Apolot D., Worodria W., Kirenga B.J., Bongomin F. (2020). Algorithm-Aided Diagnosis of Chronic Pulmonary Aspergillosis in Low- and Middle-Income Countries by Use of a Lateral Flow Device. Eur. J. Clin. Microbiol. Infect. Dis..

[22] Vergidis P., Moore C.B., Novak-Frazer L., Rautemaa-Richardson R., Walker A., Denning D.W., Richardson M.D. (2020). High-Volume Culture and Quantitative Real-Time PCR for the Detection of Aspergillus in Sputum. Clin. Microbiol. Infect..

[23] Osman N.M., Gomaa A.A., Sayed N.M., Abd A.A. (2013). Microarray Detection of Fungal Infection in Pulmonary Tuberculosis. Egypt. J. Chest Dis. Tuberc..

[24] Hedayati M.T., Azimi Y., Droudinia A., Mousavi B., Khalilian A., Hedayati N., Denning D.W. (2015). Prevalence of Chronic Pulmonary Aspergillosis in Patients with Tuberculosis from Iran. Eur. J. Clin. Microbiol. Infect. Dis..

[25] Sani F.M., Uba A., Tahir F., Abdullahi I.N., Adekola H.A., Mustapha J. (2020). Spectrum of Pulmonary Fungal Pathogens, Associated Risk Factors, and Anti-Fungal Susceptibility Pattern among Persons with Presumptive Tuberculosis at Gombe, Nigeria. Int. J. Mycobacteriol..

[26] Weig M., Frosch M., Tintelnot K., Haas A., Groß U., Linsmeier B., Heesemann J. (2001). Use of Recombinant Mitogillin for Improved Serodiagnosis of Aspergillus Fumigatus-Associated Diseases. J. Clin. Microbiol..

[27] Kampen S.C., Van Jones R., Kisembo H., Houben R.M.G.J., Wei Y., Mugabe F.R., Rutebemberwa E., Kirenga B. (2019). Chronic Respiratory Symptoms and Lung Abnormalities Among People With a History of Tuberculosis in Uganda: A National Survey. Clin. Infect. Dis..

[28] Nyunoya T., Mebratu Y., Contreras A., Delgado M., Chand H.S. (2014). Molecular Processes That Drive Cigarette Smoke-Induced Epithelial Cell Fate of the Lung. Am. J. Respir. Cell Mol. Biol..

[29] Iqbal N., Irfan M., Mushtaq A., Jabeen K. (2020). Underlying Conditions and Clinical Spectrum of Chronic Pulmonary Aspergillosis (CPA): An Experience from a Tertiary Care Hospital in Karachi, Pakistan. J. Fungi.

[30] Kreisel C.F., Passannante M.R., Lardizabal A.A. (2019). The Negative Clinical Impact of Diabetes on Tuberculosis: A Cross-Sectional Study in New Jersey. J. Endocr. Soc..

[31] Baker M.A., Harries A.D., Jeon C.Y., Hart J.E., Kapur A., Lönnroth K., Ottmani S., Goonesekera S.D., Murray M.B. (2011). The Impact of Diabetes on Tuberculosis Treatment Outcomes: A Systematic Review. BMC Med..

[32] Denning D.W., Riniotis K., Dobrashian R., Sambatakou H. (2003). Chronic Cavitary and Fibrosing Pulmonary and Pleural Aspergillosis: Case Series, Proposed Nomenclature Change, and Review. Clin. Infect. Dis..

[33] Severo L., Geyer G., Porto N., Wagner M., Londero A. (1997). Pulmonary Aspergillus Niger Intracavitary Colonization. Report of 23 Cases and a Review of the Literature. Rev. Iberoam. Micol..

[34] Agrawal M., Bajaj A., Bhatia V., Dutt S. (2016). Comparative Study of GeneXpert with ZN Stain and Culture in Samples of Suspected Pulmonary Tuberculosis. J. Clin. Diagnostic Res..

